# Recent advances in the detection and management of early gastric cancer and its precursors

**DOI:** 10.1136/flgastro-2018-101089

**Published:** 2020-07-30

**Authors:** William Waddingham, Stella A V Nieuwenburg, Sean Carlson, Manuel Rodriguez-Justo, Manon Spaander, Ernst J Kuipers, Marnix Jansen, David G Graham, Matthew Banks

**Affiliations:** 1 Gastroenterology, University College London Hospitals NHS Foundation Trust, London, UK; 2 Research Department of Pathology, UCL Cancer Institute, London, UK; 3 Gastroenterology and Hepatology, Erasmus University Medical Center, Rotterdam, The Netherlands; 4 Pathology, University College London Hospitals NHS Foundation Trust, London, UK

**Keywords:** gastric adenocarcinoma, gastrointesinal endoscopy, gastric inflammation, gastric metaplasia, Helicobacter pylori - gastritis

## Abstract

Despite declines in incidence, gastric cancer remains a disease with a poor prognosis and limited treatment options due to its often late stage of diagnosis. In contrast, early gastric cancer has a good to excellent prognosis, with 5-year survival rates as high as 92.6% after endoscopic resection. There remains an East-West divide for this disease, with high incidence countries such as Japan seeing earlier diagnoses and reduced mortality, in part thanks to the success of a national screening programme. With missed cancers still prevalent at upper endoscopy in the West, and variable approaches to assessment of the high-risk stomach, the quality of endoscopy we provide must be a focus for improvement, with particular attention paid to the minority of patients at increased cancer risk. High-definition endoscopy with virtual chromoendoscopy is superior to white light endoscopy alone. These enhanced imaging modalities allow the experienced endoscopist to accurately and robustly detect high-risk lesions in the stomach. An endoscopy-led staging strategy would mean biopsies could be targeted to histologically confirm the endoscopic impression of premalignant lesions including atrophic gastritis, gastric intestinal metaplasia, dysplasia and early cancer. This approach to quality improvement will reduce missed diagnoses and, combined with the latest endoscopic resection techniques performed at expert centres, will improve early detection and ultimately patient outcomes. In this review, we outline the latest evidence relating to diagnosis, staging and treatment of early gastric cancer and its precursor lesions.

**Key messages**

- Up to 10% of gastric cancers are missed at upper endoscopy, focus should be on quality improvement in upper gastrointestinal endoscopy.
- Chronic atrophic gastritis (CAG) and gastric intestinal metaplasia (GIM) are both precursors to gastric cancer and warrant ongoing cancer surveillance if changes are extensive.
- High-definition endoscopy with image enhancement (eg, Olympus NBI, Pentax iScan, Fujinon intelligent chromo endoscopy (FICE)) is preferred over high-definition white light endoscopy alone for the recognition of gastric premalignant conditions.
- Where endoscopic appearances suggest CAG and/or GIM, staging biopsies should be taken from at least two areas of the stomach (antrum and corpus, lesser and greater curve for each).
- Where enhanced imaging is available and with the appropriate expertise, targeted biopsies to visible mucosal abnormalities should also be taken for staging purposes.
- Early gastric cancer should be managed by referral centres with expertise in endoscopic resection. Lesions that meet standard criteria should be treated with endoscopic submucosal dissection.

## Introduction

Detection and management of gastric cancer (GC) and its precursors remain a challenge that warrants attention, and recent guidelines support an effort to make our approach in low to intermediate incidence Western countries more standardised.^1 2^ Despite declining incidence, gastric adenocarcinoma is still the fifth most common cause of cancer-related death worldwide, accounting for 8.2% of all cancer deaths.^3^ In the UK, roughly 6700 new cases are diagnosed each year.^4^ Prognosis remains poor with UK 5-year survival rates of 20.9%, and late stage of diagnosis limits treatment options in a large proportion (46%–57% with stage 4 at diagnosis).^5^ Early gastric cancer (EGC), however, has a good prognosis with a 5-year survival rate of between 69% and 82%, demonstrating the importance of early diagnosis and treatment.^6^ Importantly, a recent study showed that the incidence of non-cardia gastric adenocarcinoma is increasing among young Caucasians in the USA, and an increasing trend of atrophic gastritis in young adults has been described in Sweden.^7 8^ These data suggest that the decline in GC incidence over the past decades may be less certain in the future.

The endoscopist’s approach to upper endoscopy is a major factor in determining the success of early detection. A recent meta-analysis including 22 studies estimated a rate of missed GC at endoscopy of 9.4%.^9^ A nationwide GC screening programme in Japan contributes to earlier stage of diagnosis and with that a superior 5-year survival.^10 11^ These studies highlight the need for improved strategies to establish early diagnosis and show that the quality of diagnostic upper gastrointestinal (GI) endoscopy for the detection of neoplasia should be a target for quality improvement. Recent British Society of Gastroenterology (BSG) quality standards^2^ and newly published 2019 BSG guidelines on the diagnosis and management of patients at risk of gastric adenocarcinoma^12^ both address this. There is, however, a dearth of evidence supporting GC screening in intermediate-risk and low-risk countries. Although it has been suggested that screening programmes in intermediate-risk European countries could be cost-effective if combined with a scheduled colonoscopy^13^ or targeted to high-risk ethnic groups.^14^

Progression to (non-cardia) gastric adenocarcinoma, in the context of *Helicobacter pylori*-related chronic inflammation, results in preneoplastic transformation of the entire mucosal surface. The rugal folds and large surface area of the stomach make identification and demarcation of early premalignant lesions more challenging than in the oesophagus and colon. The time and attention endoscopists currently devote to early detection in the stomach remain far outweighed by our approach to adenoma detection in the colon. In the stomach, chronic atrophic gastritis (CAG) and gastric intestinal metaplasia (GIM) are the two main precursors that precede the development of neoplasia. Currently, the diagnosis and risk stratification of CAG and GIM are dependent on histopathology. However, improvements in advanced endoscopic imaging techniques, and an increasing body of evidence suggest enhanced imaging or virtual chromoendoscopy, can be used to reliably and accurately identify premalignant changes and indeed EGC. A shift towards an endoscopy-led staging approach in the stomach may facilitate more robust assessment to allow a more accurate and tailored approach to cancer surveillance and early detection for high-risk individuals.^15^ The advances in therapeutics in endoscopy including endoscopic mucosal resection (EMR) and endoscopic submucosal dissection (ESD) have transformed the management of EGC. ESD has become the technique of choice and is now the gold standard given its high en bloc resection rates, lower local recurrence and excellent 5-year survival rates as high as 92.6%.^16 17^ In this review, we outline the recent advances and recommended approach to endoscopic diagnosis and treatment of EGC and its precursor lesions.

## Risk factors for premalignant gastric lesions and EGC

The majority of GCs are sporadic, however, 1%–3% arise in the setting of familial cancer predisposition, including hereditary diffuse GC. This is associated with a germline mutation in the *E-cadherin* gene (CDH1) and an 80% lifetime cancer risk. Detailed guidelines describing clinical management of rarer hereditary subtypes can be found elsewhere.^18^ There are several risk factors that endoscopists should consider to assess a patient’s risk of CAG, GIM and EGC. The role of *H. pylori* in gastric carcinogenesis is long recognised,^19^ with the accepted Correa cascade describing a linear progression from chronic inflammation to atrophic gastritis, intestinal metaplasia and finally neoplasia. Serological studies suggest an underestimation of the association of *H. pylori* with CAG due to clearance of the infection in advanced stages of CAG.^20^ Patients with a history of *H. pylori* infection therefore warrant an additional degree of suspicion and mucosal inspection. Epstein–Barr virus (EBV) may play a role in the pathogenesis of a subset of gastric adenocarcinoma (up to 9%) that are molecularly distinct,^21^ however, EBV does not lead to an endoscopically detectable precursor.

Both CAG and GIM have a higher incidence in those with a family history of GC.^22 23^ Pernicious anaemia is associated with a higher risk of CAG and GIM. A recent meta-analysis of 27 studies estimated the overall relative risk in pernicious anaemia was 6.8 (95% CI 2.6 to 18.1).^24^ Advancing age is an important risk factor for gastric premalignant lesions and progression to GC. Three studies showed that patients over 45 years have an increased risk of neoplastic progression (OR 1.92–3.1).^25–27^ Multiple studies have demonstrated increased risk of CAG and GIM in male smokers^25 28–30^ and those with a high salt diet.^31^ In The Netherlands, a low incidence country, a study including patients with CAG and/or GIM diagnosed at histopathology described annual incidences of GC of 0.1% and 0.25%, respectively.^32^ Furthermore, ethnicity and geographic location appear to influence GIM-related and CAG-related cancer risk. A systematic review found higher GC incidence related to CAG and GIM in East Asian countries,^33^ while a study in the USA showed a sustained increased risk of GC in East Asian immigrants.^34^ The histological subtype of incomplete GIM may confer a higher risk of cancer progression,^35^ however, GIM is not routinely subtyped by all pathologists and further studies are warranted to establish this as an additional risk marker.

## Assessing cancer risk of premalignant lesions

Japanese data show that the grade and severity of atrophic gastritis are predictive of GC risk.^36^ However, a recent Dutch study examining surveillance in a low incidence population found that risk stratification based on biopsies alone (antrum and corpus) did not discriminate progression rate; in the low-risk group, 1 out of 86 patients developed invasive cancer compared with 2 out of 125 in the high-risk group. However, combining serology and histopathology did adequately discriminate progression risk with no patients categorised as low risk developing high-grade dysplasia (HGD) or neoplasia during follow-up.^37^ The histological Operative Link for Gastritis Assessment (OLGA) and Operative Link on Gastric Intestinal Metaplasia (OLGIM) systems have been advocated for staging gastritis.^38 39^ While higher stages of CAG and GIM by these methods (stages III and IV) are predictive of increased GC risk, current histopathologic staging methods to risk stratify these patients are all fraught with significant limitations and poor interobserver and intraobserver reproducibility.^40^ This may explain widely varying estimates of risk between studies.

**Figure 1:**
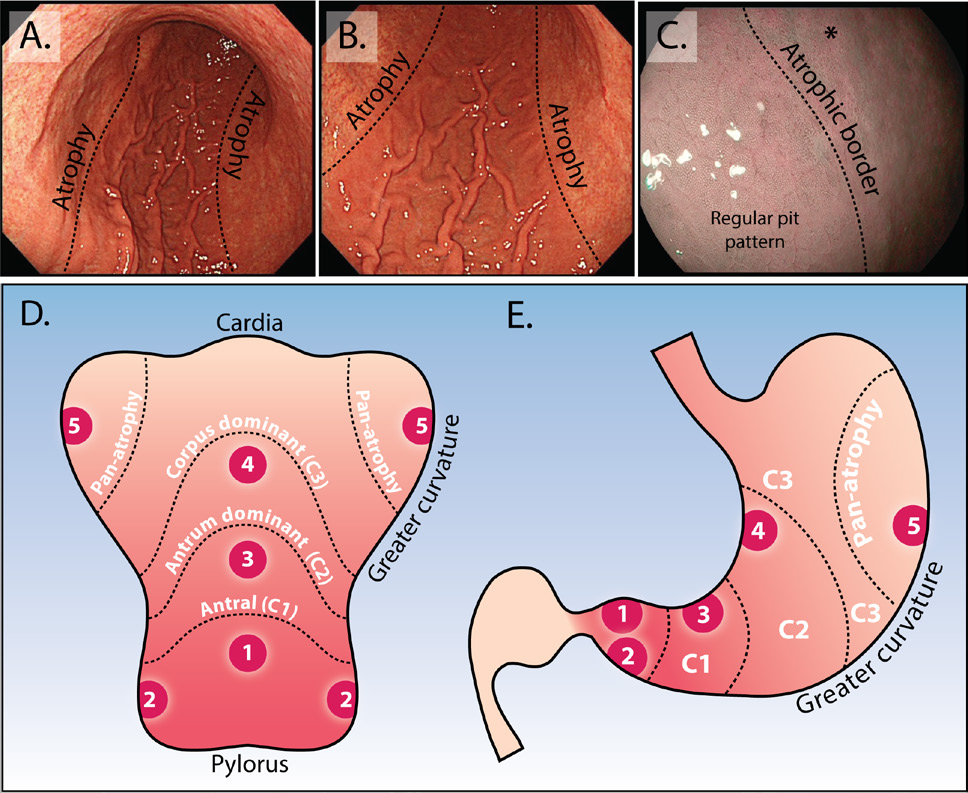
Endoscopic appearance of the atrophic border and modified Kimura–Takemoto classification system. (A and B) Low power view of atrophic gastritis at white light endoscopy. The abrupt transition at the atrophic border is clearly seen (dotted line) with loss of rugal folds, mucosal pallor and increased visibility of vessels. In this example, the atrophic border is located at the transition between the lesser and greater curve. Using the modified Kimura–Takemoto scoring system,^45^ this patient would be staged ‘C3, corpus dominant atrophy’. (C) Appearance of the atrophic border at enhanced imaging (Olympus, NBI), to the right of the dotted line the normal body pit pattern is lost and the mucosa appears paler (asterisk). (D and E) Depicted is the stomach opened up along the greater curvature (D) and in traditional coronal view (E). This schematic representation demonstrates the modified Kimura–Takemoto classification system; antral (C1); antral predominant (C2); corpus predominant (C3) and panatrophy; numbers 1–5 correspond to the location of gastric biopsies, which should be taken according to the updated Sydney system: antrum greater and lesser curve, incisura, corpus greater and lesser curve (images taken from Waddingham *et al* 15).

By contrast, longitudinal studies suggest that endoscopic staging of CAG with the Kimura–Takemoto^41^ classification system is a useful stratification tool to predict GC risk.^36 42^ This system classifies CAG into six endoscopic stages according to the location of the atrophic border. A simplified, modified Kimura–Takemoto classification is depicted in figure 1D, E.^43^ This modified system resulted in a complete concordance between endoscopic and histological assessment of 69.8% with good reproducibility (weighted kappa of 0.76 (95% CI 0.71 to 0.80)).^43^ The Endoscopic Grading of Gastric Intestinal Metaplasia (EGGIM) score is an alternative means for staging the stomach based on the presence of GIM; this relies on enhanced imaging assessment of all five areas of the stomach to score each for the presence of GIM (<30% or>30% of mucosal surface), with targeted biopsies taken to confirm the endoscopic impression.^44^ A recent validation study compared the EGGIM score with the histological OLGIM score and suggested that such an endoscopic staging system may be clinically efficacious.^44^ We strongly advocate a move towards a simplified endoscopic risk stratification system that fits within the constraints of routine (Western) clinical practice to facilitate an endoscopy-led staging paradigm to robustly predict cancer risk in the chronically inflamed stomach.

## Detection and staging of CAG, GIM and EGC

### Non-invasive assessment: serology

The best evidenced serological tests for assessing GC risk are pepsinogens. Pepsinogens secreted by gastric chief cells are the inactive proenzymes of pepsin, with hydrochloric acid leading to their conversion to pepsin. Pepsinogen I is predominantly produced in the corpus, while pepsinogen II is produced from the antrum, cardia, fundus and duodenum. As gastric atrophy progresses to the corpus, pepsinogen I is reduced relative to pepsinogen II. Therefore, a low pepsinogen I, pepsinogen I/II ratio or both are good indicators of functional atrophy. There are several studies evaluating its use in identifying patients with extensive atrophic gastritis and GC. Most recently, a 2015 meta-analysis^45^ suggested a good correlation between reduced pepsinogens and presence of gastric atrophy. The summary sensitivity and specificity for GC diagnosis were 0.69 (95% CI 0.60 to 0.76) and 0.73 (95% CI 0.62 to 0.82), respectively; corresponding values for atrophic gastritis diagnosis were 0.69 (95% CI 0.55 to 0.80) and 0.88 (95% CI 0.77 to 0.94), respectively. There were issues with study heterogeneity and varying serum cut-offs (most used pepsinogen I <70 ng/mL and pepsinogen I/II ratio <3). An American cost-effectiveness study found that one time pepsinogen screening at age 50 years reduced the lifetime intestinal-type non-cardia GC risk (0.24%) by 26.4%; however, this was not cost-effective unless targeted to current smokers.^46^ It is likely that these reasons, in addition to varying methods of laboratory serum analysis, have contributed to serological testing not being taken up in routine use in low-moderate incidence countries. Pepsinogens will likely have a future role for targeted screening of higher risk patients to identifying those who should then be offered an endoscopy.

### Endoscopy

The ESGE (2016)^47^ and BSG (2017)^2^ have listed a number of principles in their recent statements on upper endoscopy. It is recommended that a complete oesophagogastroduodenoscopy should assess and photo-document all relevant anatomical landmarks and high-risk stations, and any detected lesions. Optimal mucosal visualisation is key and should be obtained through a combination of air insufflation, aspiration and the use of mucosal cleansing techniques (eg, simethicone, N-acetylcysteine or pronase). A minimum of 7 min procedure time for first diagnostic upper endoscopy and follow-up of GIM improves detection rates of high-risk gastric lesions and is considered a key performance measure.^48^

### Endoscopic features of CAG and GIM

As atrophy progresses, the gastric rugae are lost; this combined with mucosal pallor, and increased visibility of mucosal vessels, represents the main endoscopic features of CAG.^49 50^ The atrophic border (figure 1A–C), identified as a line marking the junction of the paler atrophic mucosa and normal mucosa, which moves further proximally as the disease progresses, helps in the diagnosis of gastric atrophy and allows the endoscopist to appreciate the extent of atrophy.

**Figure 2:**
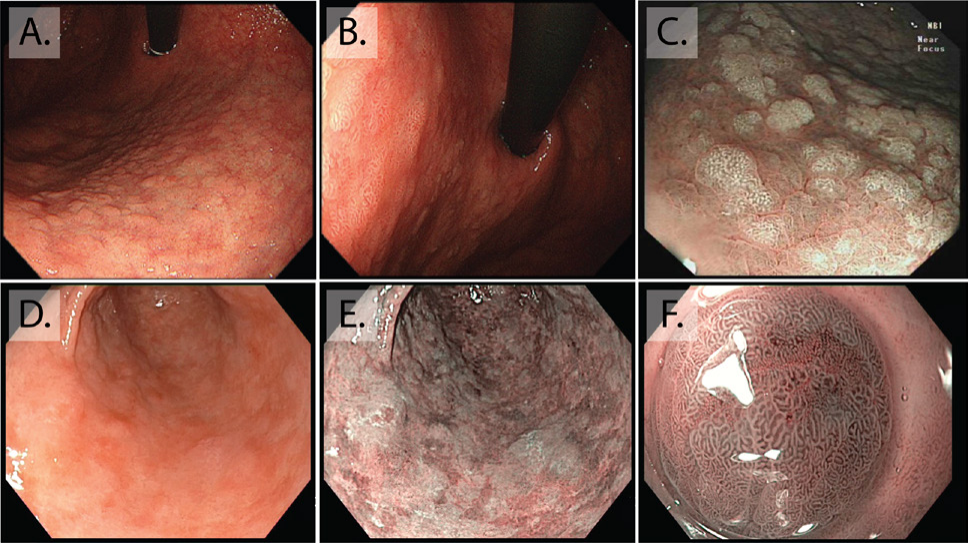
Endoscopic appearance of gastric intestinal metaplasia (GIM). (A and B) Macroscopic appearance of GIM at white light endoscopy, both are retroflexed views of the lesser curve, visible is the irregular uneven surface of GIM, with elongated groove type pit pattern. (C) At enhanced imaging (Olympus, NBI), this is visible as multiple paler elevated patches. (D and E) The difference between white light endoscopy and enhanced imaging in this stomach with extensive GIM seen as a patchwork of multiple paler, blueish patches on the background of atrophic gastritis, (F) magnification NBI allows visualisation of individual elongated metaplastic glands.

With standard white light endoscopy (WLE), GIM usually appears as paler-white, elevated plaques, surrounded by patchy pink and pale areas of mucosa causing an irregular uneven surface (figure 2). Mottled patchy erythema has also been positively associated with GIM,^51^ indeed, this could be thought to be a simple gastritis to the unwary endoscopist. Detection of GIM with standard WLE alone is of inferior accuracy compared with enhanced imaging (eg, NBI) (87% vs 53%; p<0.001)^52^ and should therefore not be used as the sole endoscopic modality.

Image-enhanced endoscopy (eg, Olympus NBI, Pentax iScan, Fujinon intelligent chromo endoscopy (FICE)) allows more detailed characterisation of the mucosal architecture. With a number of studies suggesting that superior detection rates can be achieved for both CAG and GIM.^53–56^ In the stomach, as patches of GIM expand, the glands elongate to form a ‘groove type pattern’ similar to that of the antrum or villiform pattern of the intestine (figure 2). Although these changes can easily be distinguished from the normal corpus, GIM in the antrum is more difficult to characterise.^49 57^ Additional features of GIM that can aid the endoscopic diagnosis in the antrum include the light blue crest (LBC) and the marginal turbid band.^58 59^ Using narrow band imaging (figure 2) with magnifying endoscopy (NBI-ME), the LBC appears as a fine, blue-white line on the crest of the epithelial surface and is a highly accurate sign for the presence of intestinal metaplasia at histology.^57–59^ White opaque substance (lipid droplets) obscuring the subepithelial capillaries is another endoscopic finding associated with GIM.^50^

We recommend endoscopists to routinely use enhanced imaging to make an endoscopic assessment of both GIM and atrophy. These findings should be documented with their location and extent, including the simplified Kimura–Takemoto system (figure 1D, E) (normal; limited atrophy: antral (C1); antral predominant (C2); and extended atrophy: corpus predominant (C3) and panatrophy) and finally, obtain targeted biopsies from areas endoscopically suspicious for GIM in areas of the updated Sydney protocol.^15^

### Detection of neoplasia

**Figure 3:**
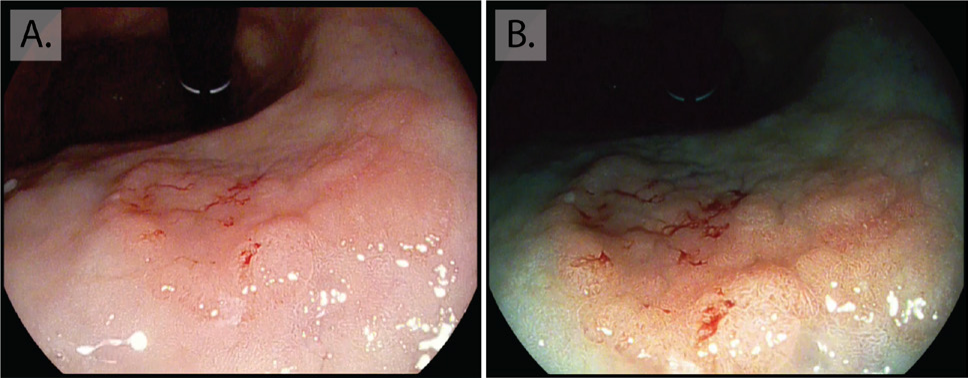
Early gastric cancer. Intramucosal cancer located in the inferior body of the stomach. Although visible at white light endoscopy (A) with nodularity, rolled edges and central depression, the lesion is more clearly demarcated with enhanced imaging (Pentax, OE) (B), showing a greater contrast of the erythematous neoplastic mucosa, irregularity in the mucosal pit pattern and loss of normal gland architecture.

High-definition endoscopy with enhanced imaging is also superior for the detection of dysplasia and early cancer. Changes in the mucosa that suggest neoplasia include irregular vessels and glands, as lesions progress this can lead to complete loss of glands and the normal mucosal and vascular pattern (figure 3). These appearances warrant photo-documentation and targeted biopsy sampling. It should be borne in mind that up to 25% of low-grade dysplasia (LGD) is upstaged after endoscopic resection. LGD lesions larger than 2 cm and those with mucosal nodularity or depression all have a higher risk of upstaging.^60^ Non-healing gastric ulcers are also a feature of neoplasia and malignancy, and multiple biopsies should be taken from the ulcer edge for confirmation, preferably targeted to areas of abnormal mucosa with irregular vascular and mucosal patterns. NBI-ME combined with conventional WLE yields a higher accuracy for detection of depressed EGCs (median 64.8%–96.6%, p<0.001).^61^ A 2016 meta-analysis confirmed that NBI-ME had a very high diagnostic efficacy for diagnosing early gastric adenocarcinoma (pooled sensitivity 0.83 (95% CI 0.79 to 0.87; I^2^=79.8%), pooled specificity 0.96 (95% CI 0.95 to 0.97; I^2^=89.3%)), this outperformed WLE although comparison was limited by study heterogeneity.^62^ Combining autofluorescence imaging with NBI has also shown very high sensitivity and specificity for dysplasia (83.33% and 98.51%) and EGC (90.91% and 99.22%).^63^ Other modalities that improve detection include blue laser imaging-bright; a Japanese randomised control trial showed that this was superior to WLE for real-time detection of EGC. Despite the high diagnostic advantage these imaging modalities afford, the majority are not widely available in Western centres and the endoscopist should therefore be advised during assessment of high-risk patients to use a combination of HD-WLE and enhanced imaging (eg, Olympus NBI, Pentax iScan or FICE) where magnification is available this can supplement the approach. The presence of atrophy and GIM should alert the endoscopist to an increased likelihood of neoplasia, initiating a more thorough mucosal assessment.

### Biopsy strategies

At first endoscopy, assessment of the high-risk stomach should include biopsies to assess for *H. pylori* and to stage the extent of atrophic gastritis.^1^ The recent 2019 BSG guidelines^12^ on diagnosis and management of patients at risk of GC recommend that patients with image-enhanced features of CAG should undergo biopsies for confirmation of endoscopic diagnosis. Biopsies should be directed at sites within Sydney protocol areas where enhanced imaging suggests GIM. Biopsy samples should be collected in separate containers and labelled as either ‘directed’ or ‘random’ to corroborate endoscopic staging.

Updated 2019 guidelines from the ESGE stipulate biopsies from at least two sites (antrum and corpus, lesser and greater curvature for each).^1^ Additionally, where enhanced imaging is available and with the appropriate expertise, targeted biopsies to visible mucosal abnormalities should also be taken. There is evidence that taking an incisural biopsy may increase the proportion of patients diagnosed with higher-risk gastritis (OLGA III/IV or OLGIM III/IV),^64 65^ this also facilitates histopathological staging with the OLGA or OLGIM, which correlate with cancer risk.^39 66 67^ Random sampling carries a significant risk of sampling error leading to inaccurate and potentially missed diagnoses; therefore, the highest yield for advanced gastritis and early neoplasia is currently with a combination of random mapping biopsies plus targeted biopsies with enhanced imaging. A drive to improve clinicians’ confidence in the recognition of mucosal patterns of the stomach, as has been the case for colonic polyp classification, would enable a move towards an endoscopy-led staging protocol where biopsies are taken for confirmatory purposes or to exclude *H. pylori* and neoplasia.

## Surveillance

### CAG and GIM

**Figure 4:**
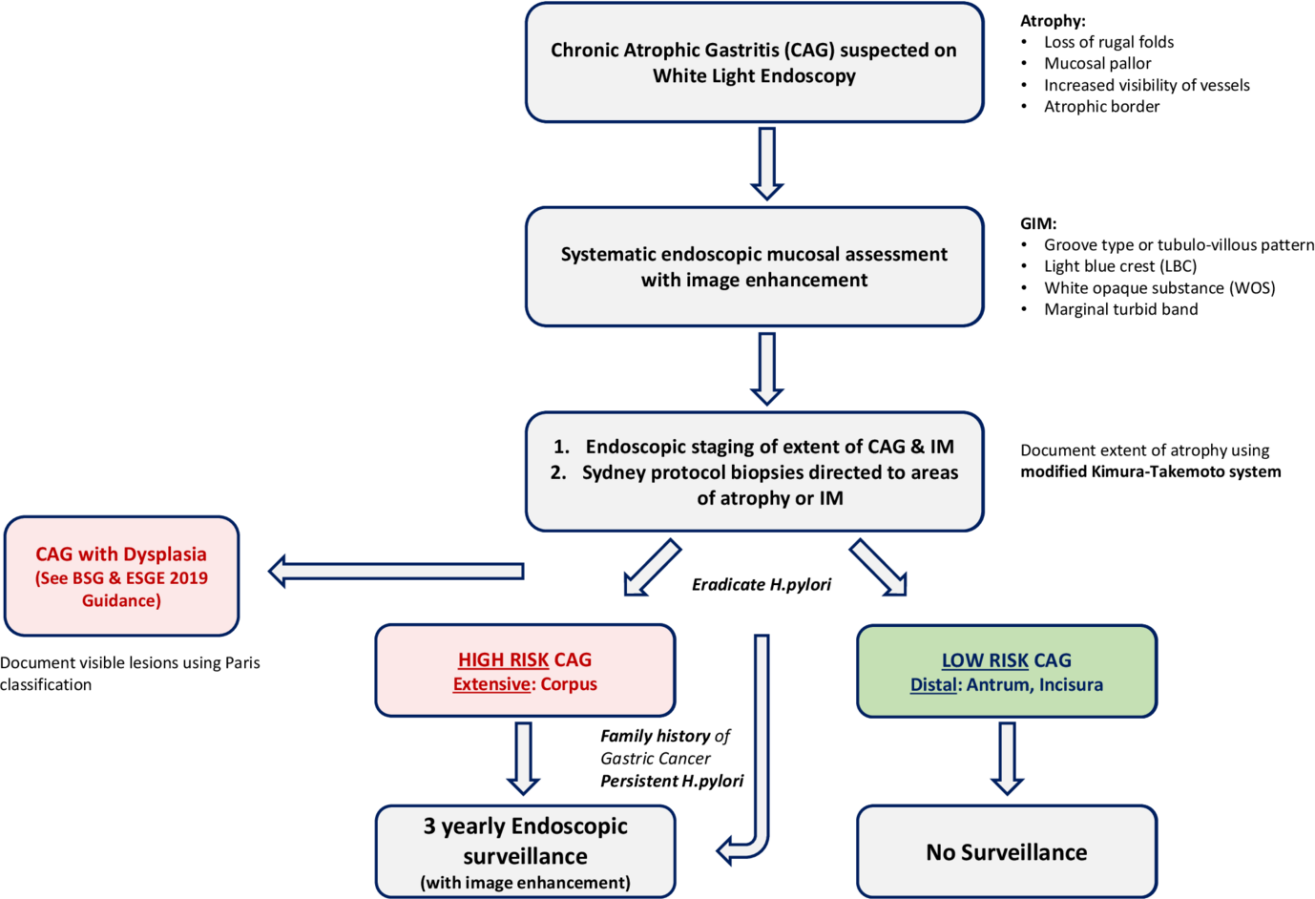
Flowchart of management of atrophic gastritis. Adapted from BSG Guidelines 2019.^12^

Recently updated European Society of Gastrointestinal Endoscopy (ESGE MAPS 2) guidelines^1 68^ and new BSG guidelines on patients at risk of gastric adenocarcinoma^12^ recommend 3 yearly surveillance for patients with extensive CAG or GIM, that is, that affecting antrum and corpus (figure 4). Although the majority of GC is sporadic, 10% show familial aggregation. Those with a family history have an additionally increased cancer progression risk, an affected first-degree relative is associated with a relative risk of 1.8–3.5.^69^ Therefore, in cases with extensive CAG or GIM and a family history, these patients should be considered for more intensive surveillance every 1–2 years, while patients with a family history and CAG or GIM limited to one area of the stomach may be counselled for the benefits of surveillance every 3 years (table 1). Carcinoma in the gastric remnant of patients who have had previous surgery for benign disease (eg, peptic ulcer disease) is rare, there are currently no consensus guidelines for this patient group; however, patients are often recommended endoscopy at 15 years post-surgery, additionally if they are known to have mucosal abnormalities such as CAG or GIM they should also be considered for surveillance on an individual basis.

**Table 1:** Summary of relevant updated guideline recommendations for diagnosis and surveillance of precancerous conditions of the stomach

Guidelines	Year	Summary of recommendations
BSG guidelines on the diagnosis and management of patients at risk of gastric adenocarcinoma.^12^	2019	**Diagnosis and staging** ‘Patients at higher risk for gastric adenocarcinoma, including GA and GIM, should undergo a full systematic endoscopy of the stomach with clear photo-documentation of gastric regions and pathology. We suggest a minimum examination time of 7 min.’ (*evidence level: moderate quality; grade of recommendation: strong; level of agreement: 100%*) ‘Patients with image-enhanced features of CAG should undergo biopsies for confirmation of endoscopic diagnosis; biopsies are directed at mucosal sites within Sydney protocol areas where enhanced imaging discloses GIM. Biopsy samples should be collected in separate containers and labelled as either ‘directed' or ‘random’ to corroborate endoscopic staging assessment.’ (*evidence level: low quality; grade of recommendation: strong; level of agreement: 93%*) **Surveillance** ‘Endoscopic surveillance every 3 years should be offered to patients diagnosed with extensive CAG or GIM, defined as that affecting the antrum and body.’ (*evidence level: low quality; grade of recommendation: strong; level of agreement: 100%*). ‘We do not recommend surveillance in patients with GA or GIM limited just to the gastric antrum unless there are additional risk factors, such as a strong family history of gastric cancer or persistent *H. pylori* infection, then we suggest 3 yearly surveillance.’ (*evidence level: low quality; grade of recommendation: strong; level of agreement: 93%*)
Management of epithelial precancerous conditions and lesions in the stomach (MAPS 2) update; ESGE.^1^	2019	**Diagnosis and staging** ‘High definition endoscopy with chromoendoscopy (CE) is better than high definition white-light endoscopy alone for the diagnosis of gastric precancerous conditions and early neoplastic lesions.’ (*high quality evidence*) ‘For adequate staging of gastric precancerous conditions, a first time diagnostic upper gastrointestinal endoscopy should include gastric biopsies both for *Helicobacter pylori* infection diagnosis and for identification of advanced stages of atrophic gastritis’. (*moderate quality evidence, strong recommendation*) ‘Biopsies of at least two topographic sites (from both the antrum and the corpus, at the lesser and greater curvature of each) should be taken and clearly labelled in two separate vials. Additional biopsies of visible neoplastic suspicious lesions should be taken.’ (*moderate quality evidence, strong recommendation*) ‘Systems for histopathological staging (eg, Operative Link on Gastritis Assessment (OLGA) and Operative Link on Gastric Intestinal Metaplasia (OLGIM) assessment) can be used to identify patients with advanced stages of gastritis. If these systems are used to stratify patients, additional biopsy of the incisura should be considered.’ (*moderate quality evidence, weak recommendation*) **Surveillance** ‘In patients with IM at a single location but with a family history of gastric cancer, or with incomplete IM, or persistent *H. pylori* gastritis, endoscopic surveillance with CE and guided biopsies in 3 years’ time may be considered.’ (*low quality evidence, weak recommendation*) ‘Patients with advanced stages of atrophic gastritis (severe atrophic changes or intestinal metaplasia in both antrum and corpus, OLGA/OLGIM III/IV) should be followed up with a high-quality endoscopy every 3 years.’ (*low quality evidence, strong recommendation*) ‘Patients with advanced stages of atrophic gastritis and with a family history of gastric cancer may benefit from a more intensive follow-up (eg, every 1–2 years).’ (*low quality evidence, weak recommendation*)

BSG, British Society of Gastroenterology; CAG, chronic atrophic gastritis; ESGE, European Society of Gastrointestinal Endoscopy; GA, gastric atrophy; GIM, gastric intestinal metaplasia; IM, intestinal metaplasia.

### Autoimmune gastritis

There is some evidence that patients with autoimmune gastritis have an increased risk of GC and may therefore benefit from endoscopic surveillance. At present, there is no clearly defined follow-up interval. Screening endoscopy at the time of diagnosis is important to secure the diagnosis but also for estimating risk as this may be the period of greatest excess cancer risk.^1^ The 2019 updated ESGE MAPS 2 guidelines recommend that patients may benefit from endoscopic follow-up every 3–5 years, however, this was a weak recommendation based on low-quality evidence.

### Dysplasia

Patients with any evidence of dysplasia should be assessed at a specialist centre with expertise in enhanced imaging assessment. All visible dysplastic lesions should be resected where possible. If there is no endoscopically visible lesion, a repeat enhanced imaging endoscopy should be performed in 6 months for HGD and 6–12 months for LGD.^1^ Revision of pathology slides by a GI pathologist with special expertise should be considered, especially in the scenario where no lesion is visible after a high-quality endoscopy. Post endoscopic resection for neoplasia, patients should remain under yearly surveillance as long as this remains clinically appropriate.

## Treatment of neoplasia

### *H. pylori* eradication

Eradication of *H. pylori* heals non-atrophic chronic gastritis, may lead to regression of atrophic gastritis and reduces the risk of GC in patients with these conditions and is therefore recommended.^1^ *H. pylori* eradication is also recommended for patients with neoplasia after endoscopic therapy. Although there is some contradicting evidence for eradication in this setting, two meta-analyses analysing 10 studies (eight non-randomised, two randomised)^70 71^ and a more recent meta-analysis analysing 17 studies reached the same conclusion that *H. pylori* eradication reduces the risk of metachronous cancer; the most recent study found a 50% lower odds of metachronous events (RR=0.50; 95 % CI 0.41 to 0.61).^72^ Subsequent to these meta-analyses, a 2018 study from South Korea, performed in a prospective, double-blind, placebo-controlled, randomised manner; again confirmed that *H. pylori* eradication after endoscopic resection for EGC lead to reduced rates of metachronous cancer (HR in the treatment group, 0.50; 95% CI 0.26 to 0.94; p=0.03) and a better chance of improvement in histological grades of atrophy (15% in placebo vs 48.4% in treatment group, p<0.001).^73^

### Endoscopic therapy

**Figure 5:**
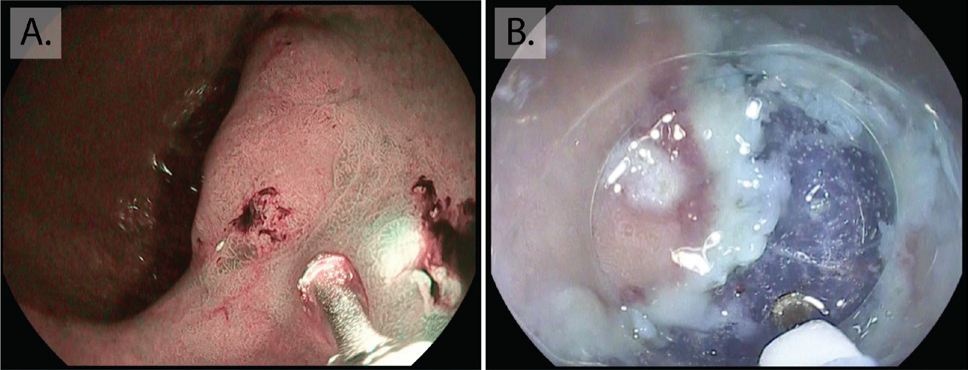
Endoscopic submucosal dissection (ESD). (A) Initial marking of a neoplastic lesion to prepare for ESD for this early gastric cancer located at the incisura. (B) ESD involves three main steps: (1) injecting fluid into the submucosa to elevate the lesion from the muscle layer, (2) circumferential cutting of the surrounding mucosa of the lesion and seen here, (3) dissection of the submucosa beneath the lesion.

The MDT plays a vital role in deciding on management of gastric neoplasia, facilitating decisions involving endoscopists, pathologists and surgeons, in centres with appropriate expertise. There is no role for endoscopic therapy in the setting of CAG or GIM; however, guidelines state that all visible gastric neoplasia should be resected in an en bloc fashion.^74^ This is in contrast to Barrett’s oesophagus where eradication of residual Barrett’s after treatment of neoplasia is recommended to reduce the risk of metachronous neoplasia. In the stomach, an ablative approach to eradicate GIM is currently neither practical nor has evidence to support it. Surveillance rather than resection for visible HGD or LGD should only be chosen if it is the patient’s preference or the risk of resection is felt to not be justifiable due to the patient’s comorbidities. ESD is the preferred technique for endoscopic resection (figure 5). Prior to performing endoscopic resection, a high-quality endoscopy should be performed with contrast or digital chromoendoscopy, by an experienced endoscopist to assess suitability (ESGE Guidelines 2015).^74^ ESD achieves significantly higher en bloc resection with lower recurrence rates than EMR and is therefore recommended by both Japanese and Western guidelines for treatment of superficial gastric neoplasia (low-grade or high-grade non-invasive neoplasia, adenocarcinoma with no evidence of deep submucosal invasion). The standard indication for endoscopic resection of gastric dysplasia and invasive cancer includes the following criteria:

- Low-grade dysplasia.
- High-grade dysplasia.
- Well or moderately differentiated intramucosal adenocarcinoma, irrespective of size and without ulceration.
- Well or moderately differentiated intramucosal adenocarcinoma, <3.0 cm in size if ulcerated.
- Well or moderately differentiated submucosal adenocarcinoma, <3.0 cm in size, with superficial submucosal invasion (Sm1; <500 micron submucosal invasion as measured in a vertical line from the deepest fibre of the muscularis mucosae).
- Poorly differentiated intramucosal adenocarcinoma, ≤2.0 cm in size

Lesions endoscopically resected with these pathological features should be considered to have been curatively treated.

## Conclusion

An awareness of higher risk patient groups combined with reliable endoscopic diagnosis and accurate assessment of the chronically inflamed stomach is essential to the early detection and successful treatment of GC. Careful mucosal examination using a combination of high-definition WLE and enhanced imaging (eg, NBI, iScan, FICE and magnification where available) should be carried out for high-risk patients. Where endoscopic signs of CAG and/or GIM are present, a combination of random mapping biopsies in the areas of the Sydney protocol and biopsies targeted with enhanced imaging provides the best chance of accurate staging and risk assessment. Gastric neoplasia should be managed by referral centres with expertise in enhanced imaging endoscopy and endoscopic resection for early cancers. Further research is needed to define the feasibility and reproducibility of an endoscopy-led staging paradigm for the premalignant stomach. This approach would help tackle the challenge of early detection, allowing more accurate risk assessment, while reducing the biopsy burden placed on patients under surveillance and on pathology services.
